# Back by popular demand, ontology: Productive tensions between anthropological and philosophical approaches to ontology

**DOI:** 10.1007/s11229-023-04243-x

**Published:** 2023-07-20

**Authors:** Julia J. Turska, David Ludwig

**Affiliations:** Knowledge, Technology and Innovation, Wageningen University and Research, Hollandseweg 1, 6706 KN Wageningen, The Netherlands

**Keywords:** Ontological turn, Philosophy of anthropology, Rights of nature, Ontological gerrymandering, Relativism, Social *ontology*

## Abstract

In this paper we analyze relations between *ontology* in anthropology and philosophy beyond simple homonymy or synonymy and show how this diagnosis allows for new interdisciplinary links and insights, while minimizing the risk of cross-disciplinary equivocation. We introduce the ontological turn in anthropology as an intellectual project rooted in the critique of dualism of culture and nature and propose a classification of the literature we reviewed into first-order claims about the world and second-order claims about ontological frameworks. Next, rather than provide a strict definition of *ontology* in anthropological literature, we argue that the term is used as a heuristic addressing a web of sub-concepts relating to interpretation, knowledge, and self-determination which correspond to methodological, epistemic, and political considerations central to the development of the ontological turn. We present a case study of rivers as persons to demonstrate what the ontological paradigm in anthropology amounts to in practice. Finally, in an analysis facilitated by a parallel between the first- and second-order claims in anthropology, and *ontology* and meta-*ontology* in philosophy (respectively), we showcase the potential for contribution of ontological anthropology to contemporary philosophical debates, such as ontological gerrymandering, relativism and social *ontology*, and vice versa.

## Introduction

There are few academic terms that create more confusion than *ontology*. While the nature of *ontology* is already deeply contested within academic philosophy (Blatti & Lapointe, 2016; Chalmers et al., 2009; Hofweber, 2018), proclamations of an *ontological turn* (Bertelsen & Bendixsen, 2017; Bessire & Bond, 2014) in social sciences and humanities further increase confusion. In anthropology, the epicenter of the proclaimed ontological turn, the promise of *ontological anthropology* has been generating widespread approval (De la Cadena & Blaser, 2018; Descola, 2013; Holbraad & Pedersen, 2017; Viveiros de Castro, 2004) or conversely critique (Fontein, 2021; Graeber, 2017; Ingold, 2016; Ramos, 2012; Todd, 2016), while increasingly also shaping debates in other areas of social sciences and humanities (e.g. DePuy et al., 2021; El-Hani et al., 2022; Furlan et al., 2020). Ontological debates in mainstream philosophy largely ignore^1^ ontological anthropology, while many proponents of ontological anthropology do not engage with philosophical literature past Deleuze and Guattari (1988) and remain disconnected from the current state of debates in philosophical *ontology*.

The aims of this article are twofold. First, we develop a diagnosis of the complex relations between *ontology* in anthropology and philosophy, arguing that they are neither homonyms nor synonyms. Second, we show how this diagnosis can foster interdisciplinary communication and dialogue. Taddei and Haines (2019) demonstrate that equivocation with respect to key concepts is a serious concern of interdisciplinary inquiry. In the case of *ontology* in anthropology and philosophy, Graeber (2017) argues that the meanings of *ontology* are drastically inconsistent, which obfuscates the understanding of what ontological anthropology sets out to do and whether in fact it delivers on any of its promises.^2^ Pace Graeber, we argue that different uses of ontology in anthropology and philosophy are not just a case of homonymy but can be related in productive ways. Instead of replacing Graeber’s claim of simple homonyms with a claim of simple synonyms, we develop a diagnosis of complex relations between meanings of *ontology* in both fields. As uses of *ontology* in anthropology and philosophy involve tangled patterns of similarity and difference, we show that these relations exhibit productive tensions that can ground interdisciplinary collaboration at the intersection of empirical and philosophical concerns.

The paper is structured in accordance with these goals as follows. We begin by introducing the ontological paradigm in anthropology and propose a distinction therein between authors who make first-order ontological claims about the world and those who make second-order claims about ontological frameworks. Based on the literature review in Sect. 2, we then proceed to conduct a conceptual analysis of *ontology* and propose a conceptualization of *ontology* in anthropology as a heuristic tool for engaging with collective-dependent, knowledge-making, self-determined interpretative domains. Afterwards, we deliberate on the parallels between the two areas of inquiry where we determine that the first- and second-order distinction can also be established in philosophical literature, specifically between ontology and meta-ontology. Based on this parallel, we analyze the tensions between philosophical and anthropological conceptions of ontology show that they can be productively explored in various areas of philosophical interest, presenting case studies of ontological gerrymandering, relativism and social ontology. In the concluding section we reflect on the relevance of bringing these conversations into dialogue.

## Ontological anthropology

Addressing the relations between *ontology* in anthropology and philosophy requires understanding of divergent uses of the term in both fields. In the anthropological literature, appeals to ontology are often grounded in a critique of a *epistemological-ontological divid*e or *Euro-Western frame* (Watts, 2013), *Western* or *modern* (Viveiros de Castro, 2012), *scientific* (Escobar, 2017)*, Northern* (Barbosa, 2022) frame, which stipulates a categorical ontological difference between the realms of Nature on one side of the binary, and Culture on the other (Vogel, 2015).^3^ The first category encompasses everything which exists independently of the human, while culture is a socially constructed branch of reality, contingently emergent from human activity. Comet is nature, poem is culture.

In literature, the orientation of authors who put forth a critique of this dualism can be coarsely divided into two groups. The first group rejects a strict division between nature and culture in favor of a different ontological order. Debates about interconnectedness and holism in Indigenous scholarship commonly put into question the separation of biological and cultural domains through emphasis of mutual dependency and ancestry (Burkhart, 2019; Chilisa, 2019; Smith, 2021; Wilson, 2008). In anthropological and philosophical debates, these ontological challenges have become reflected (Todd, 2016) in various frameworks of authors who oppose the nature/culture divide present in the European intellectual history, including Tim Ingold (2000) and his theory of perception as dwelling grounded in phenomenology and ecological psychology, and Eduardo Kohn’s (2013) ecology of selves. In Holbraad and Pedersen (2017, pp. 46–54) *alternative ontologies* is the umbrella term used to refer to this group of theories.

The second group argues that the modernist ontological framework is simply one of the many available and existing culturally contingent or collective-dependent ways of carving up reality. In this paper we refer to this strand of thought as *the pluralist ontological turn.* It maintains that the Nature/Culture dualism grounded in the assumption of a shared objective nature that is interpreted through diverse cultural lenses has dominated the field of anthropology. The division was constitutive for the most influential schools of thought throughout the history of the discipline, including interpretivism (Geertz, 2008; Martin, 1993) and cognitivism (Sperber, 1996), which both claimed that the differences between human collectives are situated at the epistemic level, i.e., at the level of the conceptual frameworks utilized and beliefs maintained by the members of these collectives. The contribution of the pluralist ontological turn consists of a shift from epistemic to ontological questions, i.e., from *what they believe* or *how they conceptualize the world* to *how the world is*, in a move which requires the shedding of a commitment to the universal applicability of the said dualist order. This interest in ontology in anthropology was sparked by the works of Marilyn Strathern (1992) and Roy Wagner (1986) on the contingency of fundamental social categories, and seminal texts include those by Descola (2013), Mol (1999) and Viveiros de Castro (2014).

As we contemplate this variety of interventions against Nature/Culture dualism, we propose categorizing them as follows. While the former group is making first-order ontological claims about the world, the latter is making second-order claims about ontological frameworks, often without explicitly committing themselves to a particular one. Inasmuch as there is this fundamental difference in how the authors produce their critiques, in the course of the literature review we have observed that there are at least three common concerns expressed throughout, regarding methodology, the episteme, and politics.

Let us start our discussion with the methodological concern. Anthropological practice is often an encounter between anthropologists and communities who differ from each other across various dimensions, including geographical location of origin, cultural background, class, gender etc. In a description of such encounters, anthropologists face the task of interpreting and communicating further a collective's point of view. Holbraad and Pedersen (2017) argue that frequently, or perhaps even inevitably, such interpretations result in a distortion of the interlocutor’s point of view, due to the incommensurability between the two ontological systems at hand.

An anthropologist, according to this view, grounds the interpretation in their own ontological framework, which leads to misrepresentation of statements, motivations, practices, or artifacts of the community in question. Moreover, while any collective can become an object of anthropological interpretation, the subject doing the interpreting is said to overwhelmingly subscribe to what we have called a modernist or Western ontological framework at the beginning of this section (Viveiros De Castro 2015). The distortion of an interlocutor’s view thereby recreates an asymmetrical power relation between the modern and the traditional, the colonial and the colonized, the Northern and the Southern, and the list goes on (Escobar, 2020; Hunt, 2013). The dominant modernist framework permeates the anthropological, and with that, renders invisible the lived reality, experience and knowledge—as well as the conceptual frameworks—of marginalized communities (De la Cadena and Blaser 2018).

This is a problem of methodological nature as far as anthropological practice goes, since it makes anthropological interpretation inaccurate at best, and completely incorrect at worst, due to the incommensurability between the frameworks of the interpreted and the interpreter. On top of that, this also raises complex epistemic issues, akin to what in philosophical literature is referred to as testimonial or hermeneutic injustice (Fricker 2019; Wanderer, 2012), which occurs “when people are maltreated in their capacity as potential conveyors of knowledge” (Wanderer, 2012, p. 148). For example, it is well-documented that Indigenous communities are experts on ecosystems they inhabit and on conservation management (Albuquerque et al., 2021) but this expertise is often framed in terms of spiritual or otherwise incommensurable perspectives that are quickly dismissed as “naive folk thinking” by scientists (Parke and Hikuroa, 2021). As a result, propositions and practices which are inconsistent with the anthropologist’s worldview are by default classified as *beliefs* as opposed to *knowledge* in the epistemic sense (Risjord, 2020). And that in turn affects how the informational output of the marginalized communities is seen as unreliable in any decision-making procedure which affects or includes diverse stakeholders, where “difference” may be seen as “mere ignorance” (Ibid., 588).

Consequently, divergences of this sort can also be analyzed on a political level when one takes into account the relationship between knowledge and power (Blaser, 2013). The dismissal and invisibility of other-than-modern frameworks is a threat to political self-determination of marginalized communities (Kramm, 2021). This political level of ontological considerations is tangibly reflected in real-world social challenges, for instance in the sphere of governance for domains such as land, water and biodiversity as described by DePuy et al. (2021). This call for ontological intervention is also seen as a way to stimulate political imagination towards thinking of radically different ways of being in the world.

## Ontology as a heuristic

In this section we address the key differences between the standard understanding of ontology in philosophy and in anthropology. By “key differences” we mean those whose discussion would enrich or clarify the debates in both disciplines. Our account is descriptive in the sense that we stay clear of mandating the *correct* use of the notion in any normative manner.

As our starting point we will take the topology of the usage of *ontology* from Graeber (2017, pp. 14–21). In his view the key difference is that in philosophy the notion refers to a theme of philosophical inquiry whereas in anthropology it is used to address frameworks which can be seen as the outcomes of such inquiries. Thus, if in philosophy ontology is in fact a set of questions, “a way of being” (ibid.), e.g., what kinds of entities exist, in anthropology it is one possible answer to those questions, e.g., naturalism of the moderns (Descola, 2013). In other words, ontology as theorizing about the nature of being versus standing for outlooks on the nature of being, respectively.^4^

Graeber’s assessment clearly captures an important use of *ontology* in philosophy (Bricker, 2016; Quine, 1953; Van Inwagen & Sullivan, 2021). However, while his definition of the anthropological sense of the term is partially consistent with the literature where the *way of being* phrasing is often used verbatim as a synonym for *ontology* (e.g. Blaser, 2013; Descola, 2013; Lloyd & Richard, 2012; Viveiros De Castro, 2015), it is analytically vague and incomplete, as it falls short in addressing the methodological, epistemic, and political considerations of ontological anthropology put forth above.

Although there is no consensus on the meaning of the term and its ambiguity is often emphasized, *ontology* is regularly described by anthropological theorists as a **heuristic** (Bond & Bessire, 2014; Holbraad & Pedersen, 2017). Given how widely, versatilely, and indeed ambiguously the term is used in anthropological analysis, we share the impression of its heuristic nature, in line with the understanding of heuristic methodologies in the humanities (Moustakas, 1990). Let us therefore unpack what it is that the heuristic of ontology in anthropology makes us understand better. Or, referring to Graeber’s terminology, how to understand *ontology* comprehensively and more precisely than as *a way of being*.

Holbraad and Pedersen (2017, p. 5) write that “the ontological turn is a response to that most fundamental anthropological question: How do I enable my ethnographic material to reveal itself to me by allowing it to dictate its own terms of engagement, so to speak, guiding or compelling me to see things that I had not expected, or imagined, to be there?”. The methodological challenge that ontological anthropology is to overcome would therefore an error in interpretation caused by applying one’s own interpretative apparatus to the ethnographic data which distorts the original meaning present therein. To mitigate this, Holbraad and Pedersen propose three methodological tools: reflexivity, conceptualization, and experimentation with respect to the analytical concepts used in ethnographic practice (Ibid.). The key takeaways from this are that the pluralist ontological anthropology is fundamentally concerned with the process of interpretation, and that this process reveals the existence of interpretative domains of the interlocutor and the anthropologist which are to be approximated by anthropological analytical concepts. Consequently, we argue that *ontology* should in this context be understood as a heuristic device used to address these **interpretative domains**.

The anthropological literature we have analyzed during this inquiry offers clues on some of the characteristics of these interpretative domains. Since across human collectives there exists a variety of these domains the research output of the discipline of anthropology speaks to, we shall add to our account of ontology the qualification that the interpretative domains we describe are **collective-dependent**. Furthermore, the process of interpretation is not per se discussed from a cognitive standpoint, and, thus, it is unclear whether these domains are seen as a concrete substrate of the process of interpretation or whether they are models of more complex processes which for analytical accessibility are labelled as domains and frameworks. On a related note, the metaphysical status of these domains is either explicitly left out of consideration as beyond the scope of anthropological interest, or even more often it is not discussed at all or discussed in an ambiguous manner; hence there is no consensus with regards to whether these frameworks consist of entities or kinds of entities, concepts or representations, practices, and so forth. To put it differently, many anthropologists reveal an agnostic attitude towards the metaphysical status of these domains. And yet the elements of the domains that are most commonly discussed are related to questions which fall under the interest of traditional metaphysics such as the composition foundational categories, for instance those of person or object, as in the analysis of the Māori concept of the *hau*^5^ by Holbraad and Pedersen (2017, p. 14, 188–189). This is where the ontological anthropology starkly diverges from the aforementioned anthropological traditions such as functionalism, cognitivism, structuralism and interpretivism, where foundational categories are seen as universal, and the task of the anthropologist is to take an established category, for instance kin, and describe how that category is conceived of in a particular collective (Palecek & Risjord, 2012, pp. 5–7). Instead, the research interest of the pluralist ontological turn focuses on reconceptualization and experimentation with the constitutive aspects of the categories themselves.

An epistemic consideration guides another aspect relevant for the overall understanding of *ontology* in anthropology. “The difficulty is that characterizing cultural differences in terms of “belief” sets up an epistemological asymmetry. The ethnographer’s problem is now one of explaining why “they” believe differently than “we” do” (Risjord, 2020, p. 593). This critique is formulated as yet again a direct departure from the above mentioned interpretative and cognitive approaches which dominated the field of anthropology since the 1980s, which positioned differences between collectives precisely at the level of belief. To address this issue, when faced with a framework which is inconsistent with one’s own, it is the view of the pluralist ontological turn that it is not sound to classify statements stemming from this worldview as beliefs, and therefore denying the reliability of knowledge that the interlocutor claims for their own statements. It is therefore paramount to recognize these interpretative domains which form the ontological heuristic as knowledge-making.^6^ From a philosophical point of view, the argument on why these claims should be interpreted as knowledge rather than belief might lack a reflection on the justification of those claims. From the perspective of anthropology, however, avoiding such normative evaluations falls within a broader tradition of “sociology of knowledge” (Berger & Luckmann, 1967) that aims to causally understand the formation of knowledge claims while often demanding symmetry between true and false believes (e.g. Bloor, 1983) in empirical treatment rather than positioning a social scientist as the ultimate authority for evaluating truth values of the contested knowledge claims.

The heuristic of ontology addresses not just methodological, epistemic, but also political concerns described in the previous section. For, according to the pluralist ontological turn, each ontology is a knowledge-making base in its own right which relates to the political concept of **self-determination** on two levels. Firstly, the formation of domains is to happen within a community which subscribes to it, and an anthropologist is to document the encountered domains as accurately as possible (e.g. Mol, 1999; Viveiros de Castro, 2014). They are also not to be revised or their validity questioned from an external point of view. Secondly, such ontology should serve as a base for decision-making processes within the realm of policy (Blaser, 2009; Kramm, 2021; Watts, 2013). In this sense, ontological self-determination becomes a core requirement for political self-determination that is affirmed by the *UN Declaration on the Rights of Indigenous People* and expressed as the ability to “freely determine their political status and freely pursue their economic, social and cultural development” (UN, 2007, p. 5) (Table 1).

**Table 1:** Conceptual analysis of the use of *ontology* in philosophy and anthropology

	Meaning of *ontology*
Philosophy	Inquiry into the nature of being
Anthropology	A heuristic tool for engaging with collective-dependent, knowledge-making, self-determined interpretative domains

Let us situate these abstract distinctions in a concrete case such as burgeoning debates about “rights of nature” (Giraldo, 2012; Santamaría Ortiz, 2023; Tănăsescu, 2020). Rivers are among the non-human entities that have become increasingly treated as legal persons with their own rights and responsibilities (Chaves et al., 2020; Kramm, 2020; O’Donnell, 2018). From the Atrato River in Colombia to the Magpie River in Canada to the Yamuna River in India to the Whanganui River in Aotearoa/New Zealand, the framing of rivers as persons is commonly motivated by local and Indigenous ontologies and their role in fostering sustainable relations between people and rivers. Recognizing “rights of nature” therefore brings together alternative ontological framings and governance practices that challenge dominant modernist perspectives on rivers as passive natural resources without agency of their own.

For philosophers, the framing of rivers as persons may raise questions about ontological commitments and their justification. If an Indigenous community considers a river a person, does that entail an ontological commitment to rivers as intentional actors? Or is there a different concept of personhood involved that does not require intentionality? For example, does the personhood of a river rely on a notion of kinship that extends to rivers because of a shared history and fate with the community? If such framings are imported from Indigenous practice into modern governance and law, does that also imply the import of Indigenous ontological commitments? Or is it sufficient to treat the personhood of rivers as a formal legal construction in analogy to the legal personhood of corporations? Finally, which of these possible interpretations involves ontological commitments that can be philosophically justified?

In contrast to such questions about ontological commitments and their justification, many anthropologists will approach personhood of rivers through the plurality of interpretative domains from the Atrato to the Magpie to the Yamuna to the Whanganui River. Such an approach focuses on **interpretative domains** in their **dependency on local collectives** rather than asking generalized philosophical questions about the justification of ontological claims about rivers. Instead of treating “a river as a person” as a suspicious belief that requires external validation, anthropologists will therefore focus on how river ontologies shape **local knowledge-making practices**. For example, the personhood of rivers may be explored in its capacity to shape relations of care and commitment between community and rivers. Such relations will be partly epistemic through situated knowledge of issues such as the river’s seasonal behavior, its capacity to support livelihood practices with fish or water, and its dangers associated with floods or rapids. The relations may also be deeply normative in the sense that they ground a moral order of relations between community and river that is radically different from modernist framings of rivers as passive objects that lack agency and need to be managed for sustainable resource extraction.

Although both anthropological and philosophical engagement with the personhood of rivers can be normative, the normativity therefore points in clearly distinct directions. While philosophers may focus on the validity and justification of ontological commitments regarding rivers, anthropological treatment of ontologies as knowledge-making heuristics leads to different forms of normativity that emphasize the **self-determination** of communities. If Indigenous river ontologies ground relations that support well-being of both communities and rivers, political ontology may approach river ontologies as part of wider political struggles for water justice instead of focusing on philosophical evaluation of ontological commitments (Boelens et al., 2022).

To summarize, we propose a conceptualization of *ontology* as a heuristic tool used in anthropology for engaging with **collective-dependent, knowledge-making, self-determined interpretative domains**. Simultaneously, in philosophy the term is largely used to address the inquiry into the nature of being.

## Comparative analysis of ontological discourses

Considering that the meanings of the key term are different, are ontological discourses under investigation developing in parallel and therefore do the exchanges amount to simple equivocations? We do not see it this way and in this section, we present an argument for why this is the case.

Distinguishing the anthropological literature along first- and second-order approaches proposed in Sect. 2. suggests a parallel with a similar division in philosophy, which experienced its own (re)turn of interest in ontology in the second half of the twentieth century (Rosen, 2014; Simons, 2004). Much of the ontological literature in contemporary philosophy is concerned with first-order ontological claims about the world. For example, extensive ontological controversies have been concerned with the existence of abstract objects (Falguera et al., 2022), composed objects (Van Inwagen, 1990), temporarily extended objects (Sider, 1997), or universals (Lewis, 1983). At the same time there has been a surge of second-order debates about ontology in philosophy. In this meta-ontological literature (Chalmers et al., 2009), pluralism has also become a widely embraced position that resembles the pluralist ontological turn in highlighting diverse ontological frameworks while avoiding commitment to any of them. In philosophical literature, meta-ontological pluralism has come to be articulated in heterogenous ways such as meta-ontological antirealism (Chalmers, 2009), neo-pragmatism (Putnam, 2004), scientific pluralism (Ludwig & Ruphy, 2021), and perspectivism (Massimi, 2021) (Table 2).

**Table 2:** First- and second-order classification of ontology-related works in philosophy and anthropology (examples)

	First-order claims about the world	Second-order claims about ontologies
Philosophy	*Ontology*	*Meta-ontology*
	Falguera et al. (2022), Lewis (1983), Sider (1997), Van Inwagen (1990)	Chalmers (2009), Chalmers et al. (2009), Putnam (2004), Ludwig and Ruphy (2021), Massimi (2021)
Anthropology	Alternative *ontologies*	Pluralist ontological turn
	Ingold (2000), Kohn (2013)	Descola (2013), Mol (1999), Viveiros de Castro (2014)

In contrast to these concerns about validity and justification in philosophy, as we have seen throughout this analysis, the ontological turn in anthropology focuses on the methodological, epistemic, and political concerns that arise from encounters between radically different collectives. Therefore, while both anthropologists and philosophers have become increasingly concerned with ontological plurality, they often approach this plurality in strikingly different ways. As we have noted, whilst philosophers tend to focus on justification and validity of ontologies, anthropologists emphasize methodological, epistemic, and political concerns that arise from inter-ontological encounters.

At the same time, we argue that the meanings of *ontology* in anthropology and philosophy are related and not just a case of homonymy. The relations we described between first- and second-order considerations open opportunities for fruitful intellectual exchange and show how debates about ontological plurality in anthropology and philosophy can be mutually enriching. The clarity with respect to the meaning of *ontology* in anthropology that our conceptual analysis in the previous section facilitates can be seen as a vehicle to engage in these cross-disciplinary debates while decreasing the risk of equivocation. In the next section we show how this strategy can be applied in practice, in order to demonstrate that the claim of entanglement of issues of diversity, interpretation, knowledge, and self-determination, heuristically addressed in the ontological turn via the concept of ontology, can bring about new developments in and across philosophical debates, and vice versa.

## The dialogue

When it comes to exchanges between philosophers and anthropologists, some topics emerge as the usual suspects, such as for instance intercultural dialogue, incommensurability, and universality. Insofar as we acknowledge the broad overlap of theoretical interests and consequently a potential for mutual inspiration between multiple debates and fields within those disciplines, in this section we will zoom in on the original insights for philosophical questions stemming distinctively from the ontological turn. While anthropologists can learn from philosophers how to embrace ontological plurality without metaphysical obscurantism, philosophers are challenged to incorporate anthropological insights about the methodological, epistemic, and political complexity of navigating ontological plurality in practice. In what follows, we sketch avenues of a possible enrichment of this sort, in the themes of philosophical consideration concerning ontological gerrymandering, relativism and social ontology.

### Ontological gerrymandering and relativism

Throughout this piece we have advocated for a closer engagement between philosophy and anthropology on the topic of ontology. One reason we have given as a motivation to strengthen this dialogue is an opportunity for philosophers to be more attentive and challenged by exploring and explaining diversity of interpretative domains. With this call for action, by no means do we claim that this variety is something philosophers have not been concerned with at all. Social constructivism, a theoretical approach which gained a lot of popularity throughout the second half of the twentieth century and whose conceptual historical lineage includes both Kant’s transcendental idealism and Marxism (Heartfield, 1996), is an excellent example of that. Social constructivism maintains that certain (or all, depending on the author) aspects of reality, such as for instance identities, are constituted by the social processes through which they emerge, rather than “naturally occurring” (ibid.). This influential idea, heavily criticized by some (Boghossian, 2007), and defended by others (e.g. Haslanger, 1995, 2003), still polarizes the philosophical and social scientific communities. In what follows we present an analysis of a prominent critique of social constructivism informed by the above considerations on ontological anthropology.

The term *ontological gerrymandering* was coined by Woolgar and Pawluch (1985) to frame an inconsistency in what they describe as the “central strategy” of the social constructivist approach to the study of social problems. In a later work, Woolgar diagnoses the core of the constructivist approach as follows:

“The key analytic logic in the social construction of social problems is the declaration that since a condition (or substance or behaviour) had not changed over a period of time, and yet the response had changed, this proved that the representation was socially contingent. For example, attitudes to marijuana smoking changed across a period of several years. Since, it is claimed, the nature of marijuana had not changed, this demonstrates the contingency of the apprehension (definition, construction). This contingency is then explained in traditional terms, through the invocation of antecedent circumstances, typically as social forces, interests and so on.” (2022, p. 180).

The ontological gerrymandering argument observes a fault in this reasoning, as therein it is left unexplained why certain features of the constructivist explanation are described as given or unquestioned, namely the stability of “the condition (or substance or behaviour)” (ibid.) over time as well as the identity of the social unit which utters the response to the condition, and some are under scrutiny and rendered as constructed such as “the apprehension (definition, construction)” (ibid.). Woolgar and Pawluch (1985) argue that this amounts to *selective relativism*, i.e., “the differential susceptibility of phenomena to ontological uncertainty” (216). While the original work was directed at social problems studies, the purchase of the argument spread to Science and Technology Studies more broadly (e.g. Gieryn, 2022), and philosophy of science and metaphysics (e.g. Kincaid et al., 2007; Travers, 2017).

In what follows, we use the equivocation-avoidant strategy outlined by this paper to explore the opportunities for mutual learnings between the inquiries into the phenomenon of ontological gerrymandering and the ontological anthropology literature. Firstly, we determine whether Woolgar and Pawluch’s argument should be situated within the realm of first-order claims about the world or the second order claims about ontological frameworks (see Sects. 2 and 4). Secondly, we sketch the implications for the narrative of the argument when ontologies are thought of anthropologically, namely as a heuristic tool used in anthropology to engage with collective-dependent, knowledge-making, self-determined interpretative domains (see Sect. 3). Finally, we spell out the avenues of enrichments stemming from those insights for both fields of inquiry, to illustrate our key claim that the tensions between philosophical and anthropological debates on ontology can be used productively.

Situating the ontological gerrymandering argument along the first- and second-order claim axis is dependent on whether one defines social constructivism as a claim about the world, in which case the argument in question is also a first-order claim, or a claim about ontological frameworks, and then the argument is a second-order claim about ontologies as well. As both interpretations are permissible by the social constructivism literature, and both offer interesting results with respect to the current investigation, we sketch out both options one by one in the remainder of this section.

Suppose that the social constructivist approach is a consequence of an ontological (in a philosophical sense) claim that the existence of some (or all) aspects of reality is grounded in the social processes which constructed this phenomenon (e.g. Haslanger, 1995), which falls in our classification under the first-order claims about the world. The binary which is created upon this formulation, between the entities which are constructed and those which are given independently, is not just the precise subject of the critique of the ontological gerrymandering argument but is also reminiscent of the Nature/Culture divide, with the natural as the given, and the cultural as the constructed, the critique of which is foundational to ontological anthropology, as described in Sect. 2. We propose that the anthropological critique of the Nature/Culture binary, foundational for interpretivism and cognitivism, could therefore be enriched by enlisting the ontological gerrymandering argument to showcase conceptual inconsistencies inherent in this ontological distinction.

The argument goes as follows. As discussed in Sect. 2., in the cognitivist and interpretivist schools of thought in anthropology, it is assumed that human beings share a set of universal biological and cognitive qualities, and that communities are situated in an environment of a material physical reality which is most accurately described by the best available natural science. These qualities and this material reality comprise Nature, the ontological realm which is governed by laws independent from human intervention. The counterpart of Nature in this ontological framework is Culture, which is everything that is contingent on the human existence, e.g., language, kinship relations, labor systems, in other words, all that is social, and therefore within the scope of social science, including anthropology. It is at this level, according to the cognitivist and interpretivist paradigms, where differences between human collectives must be explained, as, like was asserted, all residing at the level of Nature is necessary and uniform for all. However, according to the ontological gerrymandering argument, this division between the two ontological realms is itself inherently contradictory in a way that certain parts of reality are selectively placed on the side of the given, and others on the side of the contingent. And thus, assuming the Nature/Culture divide as the ontological starting point for anthropological work is not just methodologically, epistemically, politically concerning, as the ontological anthropologists argue (see Sect. 2), but on top of that is grounded on a conceptually flawed assumption of the selective binary division between Nature and Culture as shown by the ontological gerrymandering argument.

On the positive side, the purchase of alternative ontologies such as Ingold’s monistic ideas related to the phenomenologically grounded dwelling perspective (Ingold, 2000) could be expanded by showing it can also be seen as a solution to the ontological gerrymandering conundrum as follows. In Ingold’s view, the lived experience is the primary ontological category. According to phenomenological theory of experience, such lived experience cannot be divided into the given and the constructed (Lauterbach, 2018). Therefore, the ontological description in accordance with Ingold’s theory is not susceptible to the threat of gerrymandering as it removes the binary between the given Nature and the contingent Culture entirely, while still explaining the diversity of ways of being in the world by adhering to differences in lived experiences and relational networks of various collectives.

Simultaneously, the philosophical debate on ontological gerrymandering as a first-order claim about the world can be enriched through the exploration of the methodological, the epistemic and the political faces of the concept of ontology present in its anthropological definition to investigate concerns with this strategy beyond the conceptual difficulties. Arguments of this sort have been raised in other contexts outside of the ontological gerrymandering debate under the umbrella of naturalized social metaphysics (see: Porpora, 2022; Saunders, 2020). Similar intervention in the discussion under consideration could lead to investigating questions like: what are the empirical factors contributing to the maintenance of this problematic binary? Who is included and who is excluded from determining which aspects of reality should be treated as a given, and which should be questioned, or in other words, who are the agents of ontological gerrymandering? What kind of knowledge is produced and what methodologies are complicit in engaging in gerrymandering strategies? These considerations can shed new light on the phenomenon of ontological gerrymandering and consequently contribute to a broadening of philosophical debates concerning social constructivism.

To show how this works in practice, let us turn once again to the rivers as persons case studies described in Sect. 3. The status quo, when governance of water resources is concerned (DePuy et al., 2021), is that rivers are inanimate, non-agential objects belonging to the Nature ontological realm, and therefore ontologically distinct from the social realm of Culture. Despite common local dissent from this conceptualization of rivers, the binary of Nature/Culture is maintained in an entanglement of socio-political and economic factors such as for instance those surrounding large dams infrastructural sites on the Yamuna river in India (Kelkar et al., 2008). This sheds light on the selection strategies of the “selective relativism” which Woolgar and Pawluch describe, where the instrumentalist view of nature as inert allows for the exploitative activity of a kind that the Rivers as Persons view would reject. Which leads us to addressing the *who* and the *on what epistemic grounds* gets to determine the ontological status of rivers in these contexts. The implication of the works we surveyed is that state governance in tandem with the technoscientific structures and the large economic interest groups suppresses the social adherence and policy solutions grounded in the rivers-as-persons perspective to maintain the view of progress as economic development grounded in exploitation of natural resources (ibid.). The conclusion would thus be that in the case of rivers as persons, while the construction of the Nature/Culture divide is selective, as described by Woolgar and Pawluch, this process is not random, but instead generated by specific constellation of sociopolitical factors.

Until now we have focused on the interpretation of social constructivism as a first-order claim about the world. If, on the other hand, social constructivism is read as a second-order claim about ontologies (e.g. Lupovici, 2009), namely that in certain ontological frameworks, some objects may emerge via processes of social construction, then another aspect of the ontological gerrymandering concern comes to the fore, and that is selective relativism. On page 5. we have introduced the methodologies of reflexivity, conceptualization, and experimentation from Holbraad and Pedersen (2017), which represent the key strategies of the pluralist ontological turn. Each and every analytical concept employed in the analysis is supposed to be explored by the use of these methodologies, according to their prescription. Therefore, one might say, “no one is safe”. No aspect of the analysis should be treated as a given, and instead all should be radically reflexively reconceptualized and experimented with. In this way, the pluralist ontological turn overcomes the ontological gerrymandering difficulty by refusing to accept a boundary between the given and the questionable, but rather placing everything on the side of the latter.

While the intervention of second-order ontological anthropology can therefore be seen as progress in addressing the ontological gerrymandering challenge, one might ask if what this amounts to is the advocacy for what Woolgar calls “irresponsible relativism” (Woolgar, 2022, p. 180), where essentially all the interpretive domains are unstable which leads to an infinite proliferation of incommensurability in every possible configuration of collectives. Here the distinction we have proposed between first- and second-order claims comes in handy to understand without equivocation the relation between plurality claims in the pluralist ontological turn and the variety of types of relativism present in philosophical debates. The pluralist ontological turn in anthropology does not easily fit philosophical definitions of relativism as a first-order claim, such as “irresponsible relativism” Woolgar alludes to, as it largely relates to relativism as a methodological project in anthropology and other social sciences such as the “empirical programme of relativism” (Collins, 1981) in sociology of science itself, just like the pluralist ontological turn, a second-order claim about ontologies.

This relation between anthropological and philosophical approaches to relativism turns out to be productive for both sides. The empirical results of ontological anthropology present evidence for the diversity of interpretative domains which are not only linguistic, but they are also deeply entangled with practices, and epistemic and political dynamics. This complexity makes the ontologies anthropologists talk about irreducible to conceptual schemes, and so deep diversity described by the pluralist ontological turn cannot be readily dismissed based on Davidson’s renowned argument against conceptual relativism (Davidson, 1973; Palecek & Risjord, 2012), as Davidson’s argument addresses primarily the translatability of translatability between languages. Aside from rendering the existing philosophical concepts relating to diversity as lacking certain levels of complexity, this entanglement of interpretation, episteme, and politics, can be explored in its own right, fueling the existing discussions on pluralism across many philosophical domains, as exemplified by the rights of nature and rivers as persons case studies presented in Sect. 3. A lot can be learned about the relation of ontologies with practices including livelihood practices and sustainable engagement with environments (e.g. Stensrud, 2016) to develop a metaphysical understanding of a human being as one of the agents in the environmental networks rather than a key stakeholder hierarchically placed above all the other participants of the lived reality. About the political dynamics through which ontologies become dominant in practice (e.g. De la Cadena, 2019), and about how marginalized ontologies can become forms of political resistance (e.g. Hunt, 2013), to reflect deeply about the entanglement of ontology, no longer exclusively understood as a necessary given outside of the social realm, and power.

While the pluralist ontological turn is primarily grounded in methodological (second-order) rather than metaphysical (first-order) claims, some argue that they cannot be neatly separated and isolated from each other (Ramos, 2012). Political ambitions of ontological anthropologists in particular limit the anthropological suspension of judgment and ultimately require an unapologetically normative approach to ontological plurality. Here, the productive tension between anthropological and philosophical ontology provides anthropologists with normative resources from meta-ontological traditions such as perspectivism, pragmatism, and scientific pluralism that can respond to accusations that the pluralist ontological turn leads to philosophically obscure claims about multiple worlds (Graeber, 2017).

### Social ontology

In the previous section we have investigated the applicability of our methodology of bringing together conversations on ontology in philosophy and anthropology to explore the themes of ontological gerrymandering and relativism. We now move on to expanding our consideration to another field of philosophical interest, namely social ontology, and thus present productive tensions and opportunities for enrichment between the two disciplines at a larger scale.

Philosophical ontology is undergoing a process of transformation and of broadening of research agendas. While analytic metaphysics of the second half of the twentieth century remained detached in its focus on fundamental entities such as universals or abstract objects, the emergence of social ontology reflects an increased philosophical concern with the social domain (Barnes, 2014; Ludwig, 2021). As a relatively novel field, social ontology can be interpreted in a variety of ways. First, social ontology can be defined through its concern with the ontology, in its philosophical sense as an inquiry into the nature of being, of the social world. In this way, it aligns with our classification as a first-order investigation. Along these lines, Baumann and Bultmann (2020, p. 1) describe the core interest of social ontology as “the most encompassing and most deeply embodied meaningful structures that define what collectives are, how they structure themselves, and which entities can become part of them”. In this sense, the scope of social ontology is defined by the social world—e.g., it includes questions about the ontology of social groups and institutions in general terms but also particular social entities such as gender or money. While ontological anthropology is partly concerned with the social world, it is focused on the interpretative domain of a collective which often reaches beyond the social world as reflected in ontological conflicts about entities such as forests, jaguars, or rivers.

Second, social ontology can also be interpreted as being concerned with the social dimensions of ontological negotiations rather than exclusively as the ontology of the social world, and thus engaging with second-order claims about ontologies. While the first interpretation defines social ontology through its domain of application (the social world), the second interpretation expands the considered dimensions of ontological negotiation (social, rather than exclusively epistemological or metaphysical). This second interpretation positions “social ontology” in similar ways as “social epistemology”. Social epistemologists are not merely concerned with knowledge about the social world but rather with the social dimensions of producing and negotiating knowledge. For example, social epistemologists do not only discuss knowledge production in the social sciences but have also critically engaged with epistemic practices in biological and other natural sciences (Wagenknecht, 2016; Wray, 2011). In analogy, a broader reading of social ontology also opens space for reflexivity about the social dimensions of producing and negotiating ontological frameworks—no matter whether in social sciences, biological sciences, or some other domain.

Ontological anthropology provides rich empirical and methodological resources for addressing this second issue of the social dimensions of ontological negotiations. Many social ontologists have challenged the idea of value-freedom in ontology (Díaz‐León, 2021; Ludwig, 2016; Mikkola, 2015), pointing out that social values are deeply entangled with ontological frameworks. Ontological anthropology allows to explore this entanglement via its heuristic grasp on collective-dependent, knowledge-making, self-determined interpretative domains. Rather than simply state that social values and ontological frameworks shape each other, ethnography provides an entry point for understanding how this entanglement is constituted in practice. Consider prominent cases that are discussed in ontological anthropology such as thinking forests (Kohn, 2013) or rivers as persons (Kramm, 2021). Ethnographic methods help to understand how ontological and moral orders are intertwined and shape each other. Ontologies that expand agency to non-human entities such as forests and rivers shape moral reasoning just as Indigenous norms of engaging with non-human entities shape their ontological framing. In this sense, many Indigenous communities embrace ontological and moral orders that fundamentally differ from current sustainability frameworks which reflect modern economic thinking about rivers and forests as finite natural resources that need to be exploited in non-destructive ways but are not moral agents in their own right.

Understanding social dimensions of ontological negotiation requires contextual and empirical depth while purely philosophical discussions of ontology tend to be abstract and generic. Integrating conceptual tools from philosophy with ethnographic depth from anthropology provides a much richer entry point for engaging with these complex realities of ontological negotiation. One of the clearest examples of this is philosophical reflexivity about the political dimensions of ontological plurality. Ethnography is key for understanding how different ontologies do not only constitute abstract possibilities of carving up the world but that they rather guide action and practice. Ontological anthropology shows how local ontologies support local livelihood practices from farming and fishing to community organization. Ethnography and interpretative methods such as reflexivity, conceptualization, and experimentation (Holbraad & Pedersen, 2017) are also crucial to understanding inter-ontological encounters and the marginalization of Indigenous ontologies in processes of development and modernization (Escobar, 2017). For social ontology, these issues constitute an important opportunity to overcome its narrow and often deeply Eurocentric focus by opening ontological perspectives on global negotiations of livelihoods, modernization, and development. Careful engagement with insights from ontological anthropology therefore constitutes an invitation to social ontologists to develop a broader and practice-oriented approach towards the interface of ontology and society.

## Being with difference

“Anthropology … is akin to philosophy …, but differs from philosophy (at least as practiced by the majority of professional philosophers) in that it does its philosophizing in the world, in conversation with its diverse inhabitants rather than in arcane reflections on an already established literary canon” (Ingold, 2017, p. 24). It is becoming increasingly recognized that philosophy needs to be engaging with the material issues of practice and empirical reality of plurality. This paper emerged from our experience as scholars of “philosophy of science in practice” (Ankeny et al., 2011; Boon, 2017) involved in an interdisciplinary research project focusing on the relation between metaphysics and the practical dimension of knowledge diversity.^7^ In the course of our research, we became aware of the scale of interest^8^ in ontology in the context of social difference in the social sciences, and anthropology in particular. Yet early on we came to realize that while the input of the anthropological investigations of this topic was very relevant to our own work, the usage of the term *ontology* in these texts was far from clear. Therefore, to meaningfully engage with this literature, we had to understand how this ontology relates to philosophical ontology, and the current work is the result of this investigation.

This article therefore aims at conceptual clarification, aligning related but distinct uses of *ontology* in different academic communities. At the same time, our ambitions reach beyond conceptual clarification, aiming to contribute to new spaces of interdisciplinary exchange. An exchange which, strengthened by the said clarification and the strategy outlined here, can proceed while the risk of equivocation is minimized, as exhibited in our analyses of ontological gerrymandering, relativism and social ontology. We see this result as important, as the prevailing silence between anthropological and philosophical research communities only amplifies confusions and limitations of academic debates about ontology.

Philosophical engagement with ontology has substantially broadened in scope as most clearly reflected in the burgeoning literature on social ontology and human kinds (Haslanger, 2012; Jenkins, 2020; Mikkola, 2015). At the same time, philosophers often lack methods and training to navigate the empirical complexity of ontological contestations. While experimental philosophy has mainstreamed some empirical methods in philosophy (Kiper et al., 2021), experimental and quantitative approaches alone are not sufficient enough to navigate heterogeneous practices and politics of ontological negotiations on a global scale. The ontological turn in anthropology provides philosophers with a unique opportunity to overcome these limitations through ethnographic depth that takes ontological plurality seriously in its entanglement with lives and livelihoods of diverse people. For philosophers, surmounting the current disconnect with anthropologists constitutes a major opportunity to expand their methodological toolbox and to align it with the broadening scope of philosophical ontology in the social domain. We are hopeful that this piece, where we apply traditional analytic philosophy methodologies such as literature review and conceptual analysis to investigate works of anthropologists and search for connections with philosophical debates on ontology, can contribute to facilitating progress in this aspect.

For anthropologists, a dialogue with philosophers also comes with opportunities of addressing current limitations and frustrations. While the ontological turn is testimony to innovative conceptual experimentation in anthropological theory, it also reflects confusion and ambiguities that result from the anthropological proliferation of theoretical concepts. When Kohn (2013, p. 139) asks “Can anthropology make general claims about the way the world is?”, anthropologists appear to give radically different answers—from positioning themselves as metaphysicians who make first-order claims about the world to emphatically denying any metaphysical ambitions. But even if the ontological turn is carefully constructed as a methodological rather than metaphysical project in line with the view of ontology as a heuristic device we present here, it inevitably bumps into thorny epistemological, metaphysical, and normative questions about ontological plurality. Rather than pretending that anthropologists can thoroughly isolate themselves from such philosophical questions, this article constitutes an invitation for both parties to enter into a mutually enriching dialogue that remains sensitive to different conceptual legacies of *ontology* in anthropology and philosophy.

## Footnotes

1 This promise of a new ontological paradigm in anthropological research is a point of departure for authors with philosophical interests ranging from relativism (Koskinen, 2019; Palecek & Risjord, 2012), immanence and temporality (Bond & Bessire, 2014), de-colonial epistemology (Ludwig, 2018; Nunes, 2009), to the critique of representationalism (Risjord, 2020). It is also contributing to a growing recognition of “global epistemologies and philosophies of science” (Ludwig et al., 2021) in the mainstream of academic philosophy. Nevertheless, ontological debates in academic philosophy remain largely isolated from this career of *ontology* across social sciences and humanities.

2 We discuss Graeber’s argument in more detail later in the paper (Sect. 3), where we re-examine his conceptual analysis of ontology in anthropology and propose a revised conceptualization of how the term is used.

3 For the purpose of this paper, we leave aside the question of whether these labels, specifically those alluding to a geographical location, such as *Western* or *Northern*, are adequate in describing this specific dualist ontological order, see Allais (2016).

4 Graeber (2017, p. 18) also argues that *ontology* in the philosophical sense falls under how *epistemology* is used in anthropology, where *epistemology* in the anthropological sense is a *way of knowing*, i.e., ontology as a philosophical inquiry is a particular type of a knowledge production practice which is called an *epistemology* by anthropologists.

5 Hau, “a cosmic power and a vital essence” (Hēnare, 2015, p. 89), is a much-discussed concept in social and cultural anthropology, often in relation to the work of Mauss (1990) on gift exchange.

6 The phrasing *knowledge-making* is used here to leave it undetermined whether ontology should be understood as a system of knowledge itself or as knowledge-grounding, which would depend on the metaphysical outlook on the relation between knowledge and the entities it describes. This issue is not sufficiently described in literature to unequivocally offer one interpretation over the other. While further discussion of this is beyond the scope of this paper, what we want to emphasize is that in terms of epistemic concepts, ontology in anthropology should be understood as related to knowledge, not belief.

7 https://www.geos-project.org/

8 “Drawing on Google Scholar one can see that between 1960 and 1990 there were only eight articles published which had anthropology and ontology—related words in the title, while between 1990 and 2016 the number was approximately 90. And akin to the ontological maneuver of a reversal of perspectives, our impression is that these 90 merely comprise the tip of the proverbial iceberg.” (Bertelsen &Bendixsen, 2017, p. 1).
